# AGING AND RADICAL ENVIRONMENTAL CHANGE: “WHY DON’T THEY JUST LEAVE?”

**DOI:** 10.1093/geroni/igae098.4117

**Published:** 2024-12-31

**Authors:** Marlene Friis, Jeanette Gustat, Katie Cherry, James Cronin, William Bertrand

**Affiliations:** Tulane University, New Orleans, Louisiana, United States; Tulane University School of Public Health and Tropical Medicine, New Orleans, Louisiana, United States; Louisiana State University, Baton Rouge, Louisiana, United States; XXX; Tulane University, New Orleans, Louisiana, United States

## Abstract

Coastal Louisiana has experienced extensive land loss due to oil and gas extraction, flood protection measures, and repeated exposure to hurricanes, flooding, and other severe weather events. Radical socioeconomic changes further negatively impact older residents. Projected relative sea-level rise, increased intensity of weather events, and large infrastructure projects may further alter the ecosystem, leaving residents with the option to either adapt or retreat. Governmental agencies are working towards reducing population vulnerability through risk reduction measures, disincentivizing further population growth, or promoting outmigration to higher ground. Critically, older adults may be uniquely vulnerable to the hazards associated with coastal living as they are both overrepresented in population statistics yet may also be less sensitive to policies designed to move people out of harm’s way. Regarding migration decisions, older adults are often deeply embedded in the socioecological systems of historic communities and typically value the benefits of continuity over disruption. Using the Environmental Distress Scale on a sample of geographically vulnerable adult residents in lower Plaquemines Parish, Louisiana (n=161) and in-depth interviews with older adults (n=45), results indicate that Place Attachment, Solastalgia, Threat Perception, Felt Impact, and Action may undergird decisions to stay put in the face of radical environmental change. Results show that most adults ages 55 and over (75%) intend to stay in the area. Although some older adult may qualify as trapped (involuntarily immobile), this research supports older adults’ agency in choosing to be voluntarily immobile in the face of slow-onset environmental changes. This distinction has policy implications.

